# Localizing hierarchical prediction errors and precisions during an oddball task with volatility: Computational insights and relationship with psychosocial functioning in healthy individuals

**DOI:** 10.1162/imag_a_00461

**Published:** 2025-02-03

**Authors:** Colleen E. Charlton, Daniel J. Hauke, Michelle Wobmann, Renate de Bock, Christina Andreou, Stefan Borgwardt, Volker Roth, Andreea O. Diaconescu

**Affiliations:** Krembil Centre for Neuroinformatics, Centre for Addiction and Mental Health (CAMH), Toronto, ON, Canada; Centre for Medical Image Computing, Department of Computer Science, University College London, London, United Kingdom; Department of Psychiatry (UPK), University of Basel, Basel, Switzerland; Department of Psychiatry and Psychotherapy, Translational Psychiatry, University of Lübeck, Lübeck, Germany; Department of Mathematics and Computer Science, University of Basel, Basel, Switzerland; Institute of Medical Sciences, University of Toronto, Toronto, ON, Canada; Department of Psychiatry, University of Toronto, Toronto, ON, Canada; Department of Psychology, University of Toronto, Toronto, ON, Canada

**Keywords:** sensory learning, hierarchical Gaussian filter, predictive coding, EEG, functioning

## Abstract

The auditory mismatch negativity (MMN) has been widely used to investigate deficits in early auditory information processing, particularly in psychosis. Predictive coding theories suggest that impairments in sensory learning may arise from disturbances in hierarchical message passing, likely due to aberrant precision-weighting of prediction errors (PEs). This study employed a modified auditory oddball paradigm with varying phases of stability and volatility to disentangle the impact of hierarchical PEs on auditory MMN generation in 43 healthy controls (HCs). Single-trial EEG data were modeled with a hierarchical Bayesian model of learning to identify neural correlates of low-level PEs about tones and high-level PEs about environmental volatility. Our analysis revealed a reduced expression of the auditory MMN in volatile compared to stable phases of the paradigm. Additionally, lower Global Functioning (GF): Social scores were associated with a reduced difference waveform at 332 ms after stimulus presentation across the entire MMN paradigm. Further analysis revealed that this association was present during the volatile phase but not the stable phase of the paradigm. Source reconstruction suggested that the association between the stable difference waveform and psychosocial functioning originated in the left superior temporal gyrus. Finally, we found significant EEG signatures of both low- and high-level PEs and precision ratios. Our findings highlight the value of computational models in understanding the neural mechanisms involved in early auditory information processing and their connection to psychosocial functioning.

## 1 Introduction

Deficits in early auditory information processing, as measured by event-related potentials (ERP), provide critical insights into the pathophysiology of psychosis and its connection to psychosocial functioning (Ro & Clark, 2009;Thomas et al., 2017). The mismatch negativity (MMN), an ERP component reflecting the brain’s capacity to automatically detect and encode novel or unexpected auditory events, has been especially useful in studying automatic sensory processing and its relationship to psychosocial functioning (Friston, 2005;Garrido, Kilner, Stephan, et al., 2009;Näätänen et al., 2007). Notably, reductions in the MMN, along with the subsequent P3a component, have been linked to reduced psychosocial functioning in healthy adults (Light et al., 2007) and across the psychosis spectrum (Carrión et al., 2015;Hermens et al., 2010;Murphy et al., 2020).

Recent advances in computational neuroscience, particularly within the predictive coding framework, have reframed our understanding of the MMN as a reflection of prediction error (PE) processing in the brain (Friston, 2003,^2005^;Garrido, Kilner, Stephan, et al., 2009;Lieder, Daunizeau, et al., 2013;Rao & Ballard, 1999). The MMN is thought to reflect the brain’s inability to suppress PEs in response to unpredictable sensory input. This framework emphasizes the hierarchical nature of information processing, where higher-level PEs encompass abstract, slowly changing information, while lower-level PEs focus on rapidly changing basic sensory features (Kiebel et al., 2008;Wacongne et al., 2011).

The hierarchical Gaussian filter (HGF), a hierarchical Bayesian model of learning, illustrates this concept by modeling the impact of PEs through precision-weighted updates (Mathys et al., 2011,^2014^). In this framework, the precision ratio serves as a learning rate that influences the balance between prior beliefs and new sensory input. Modulation of the precision ratio is thought to involve neuromodulatory gain control mechanisms, possibly involving N-methyl-D-aspartate (NMDA) and acetylcholine receptors, which encode precision as changes in neuronal excitability (Kanai et al., 2015;Schöbi et al., 2021;Weber et al., 2022). In stable settings, high precision is assigned to our prior beliefs, minimizing the impact of sensory fluctuations. In volatile environments, the precision ratio (i.e., gain control) shifts to prioritize lower-level sensory inputs, ensuring effective adaptation to environmental changes.

While much research has focused on the detection of the MMN at the sensor level, this approach provides limited insight into the specific cortical regions involved in generating and modulating PEs. Based on PE accounts of the MMN, we predict that low-level PEs are generated in early sensory regions (Parras et al., 2017), whereas high-level PEs are computed in higher-level regions, including the inferior frontal gyrus (IFG) (Camalier et al., 2019). Localizing the neural sources of PEs is crucial for identifying how different cortical areas contribute to predictive processing and interact during auditory learning, enabling non-invasive analysis of brain dynamics (Asadzadeh et al., 2020).

Prior research has demonstrated the sensitivity of the MMN to environmental volatility, showing larger MMN amplitudes in stable conditions, where deviant tones are less expected, and smaller amplitudes in volatile settings where deviant occurrences are more frequent (Dzafic et al., 2020;Kotchoubey, 2014;Todd et al., 2011,^2014^;Weber et al., 2022). Several of these studies used the local-global paradigm to show how changes in MMN amplitude are linked to variations in the regularity of auditory stimuli. This paradigm distinguishes between local (tone-to-tone transitions) and global (sequence-to-sequence transitions) regularities, suggesting that local-rule violations are processed in lower-order cortical areas, whereas global-rule violations are detected by higher-order areas (Fong et al., 2020).

Building on these findings, we employ a modified auditory oddball paradigm (Weber et al., 2022) with alternating stable and volatile periods to better distinguish learning about tone probabilities and environmental volatility. The manipulation of volatility is crucial as it allows us to explore how the brain dynamically adjusts its predictive processes in response to environmental changes. In classic oddball paradigms, low-level and high-level PEs are often correlated (Charlton et al., 2022). Our paradigm is deliberately designed to decorrelate low-level and high-level PEs to achieve a clearer separation between these processes. Additionally, manipulating volatility broadens the range of environmental dynamics participants must learn about, extending beyond the fixed deviant probabilities seen in the MMN paradigm. This approach allows us to examine how the brain flexibly adjusts its predictive mechanisms across a wider spectrum of true volatility, offering new insights into dynamic learning processes.

Unlike the local-global paradigm, which primarily examines “what” is changing (i.e., local or global deviance), our study uses the HGF, which examines “how fast” these auditory changes occur at different hierarchical levels. The HGF assumes a specific relationship between hierarchically-coupled states that evolve in time as Gaussian random walks, linked through their variances (Hauke, 2022). This approach allows for a more nuanced and dynamic perspective of change, beyond the static learning process often assumed in classical MMN analyses.

In our exploratory study, we employ a computational approach using the HGF to model single-trial electroencephalogram (EEG) data in a novel MMN paradigm. This approach diverges from traditional MMN analyses, which typically assess amplitude changes from a limited selection of trials. By modeling brain activity on a trial-by-trial basis with the HGF, we can capture intermediate learning signals for a more accurate depiction of the brain’s continuous and dynamic learning processes. We investigate the individual components of precision-weighted PEs—precision ratios and unweighted PEs—and examine their associations with psychosocial functioning in the general population. Furthermore, we use multiple sparse prior (MSP) source reconstruction (Friston et al., 2008) to localize the cortical generators of PEs and their precision weights, offering insight into the interactions between brain regions during predictive coding. By integrating the HGF model with MSP source reconstruction, our exploratory study aims to provide a more comprehensive understanding of MMN generation and its relationship with psychosocial functioning.

## 2 Methods

### 2.1 Participants

A total of 43 healthy controls (HC) were recruited through online and public advertisements in Basel, Switzerland. HCs were recruited as part of a larger study investigating persecutory ideation in early psychosis, described in greater detail elsewhere (Hauke et al., 2024). Due to insufficient patient data, only results from HCs are presented here. Inclusion and exclusion criteria are detailed in theSupplementary Material. All participants provided written informed consent, and the study was conducted in accordance with the Declaration of Helsinki and approved by the local ethics committee (Ethikkommission Nordwest- und Zentralschweiz, no. 2017-01149).

### 2.2 Demographic variables and functioning assessment

Demographic and functioning data were collected during an interview within 5 days of EEG data collection. Functioning was assessed with the Global Functioning: Social (GF: Social) and the Global Functioning: Role (GF: Role) scales (Cornblatt et al., 2007). The former measures social relationships and interpersonal activities, and the latter assesses performance and functioning in work, education, and home activities depending on the age of the individual. A single score for each scale was provided by trained raters, with higher scores indicating better performance. The median score for GF: Social was 9 and for GF: Role was 9, with both scores ranging from 6 to 10. Demographic characteristics are summarized inTable 1.

**Table 1.** Participant characteristics.

	Healthy controls
Age (mean [SD])	22.9 [6.8]
Years of education (mean [SD])	13.2 [3.3]
Working memory (mean [SD])	6.6 [2.1]
Sex (f/m)	20/23
Handedness (l/r)	5/38
Cannabis (y/n)	27/16

Demographic characteristics of the study sample.

### 2.3 Mismatch negativity paradigm

**Fig. 1.**
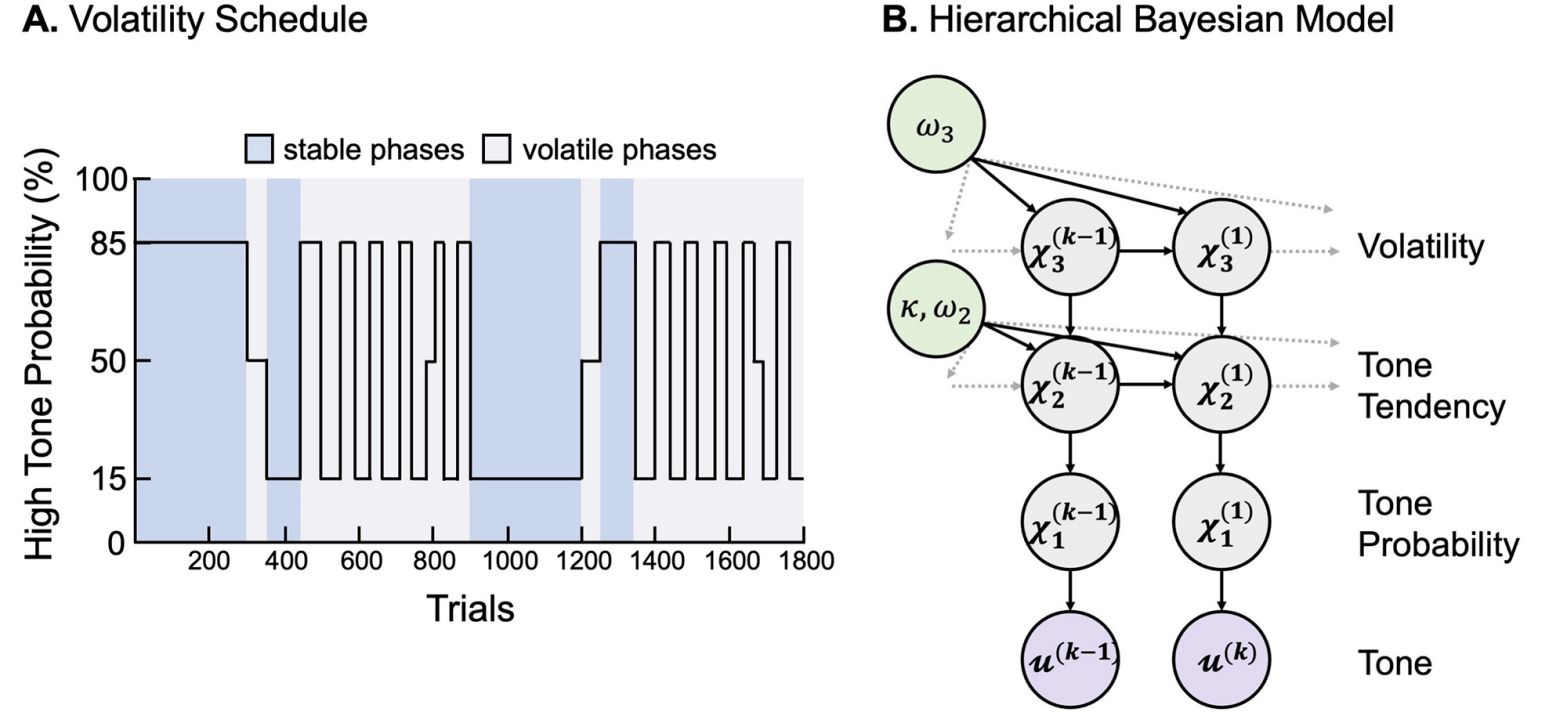
Mismatch volatility schedule and computational model. (A) Volatility schedule of mismatch negativity paradigm. (B) Three-level hierarchical Gaussian filter (HGF) binary perceptual model.

EEG data were collected during the mismatch negativity paradigm specifically designed to minimize the correlation between low-level and high-level precision-weighted prediction errors (for details, seeWeber et al., 2022). As such, the task included periods of volatility, characterized by rapid changes in the probabilities of the tones, and periods of stochasticity, where the tone probability averaged at 50%. Participants were presented with a series of tones delivered binaurally through Etymotics HF5 headphones, while they engaged in a visual distraction task to direct participants attention away from the tones (seeSupplementary Materialfor more details). The auditory stimuli comprised two pure sinusoidal tones: a high (528 Hz) and a low (440 Hz) tone, each lasting 70 ms (including 5 ms fade-in/fade-out) with an inter-stimulus interval of 500 ms and totaling 1800 tones. Stimuli were presented at comfortable loudness determined by the participants. The task consisted of two types of phases: stable phases were defined as periods where the probability of hearing the same tone remained constant for at least 90 trials and volatile phases were all other phases (Fig. 1A). Auditory and visual stimuli were presented using PsychToolbox (PTB3, psychotoolbox.org; version 3.0.14) and Matlab (R2018a).

Participants’ responses during the visual distraction task were analyzed by computing mean reaction times and hit rates, which are reported in theSupplementary Material. One subject had a hit-rate below 75%, which may suggest that the individual was attending the tones instead of the visual task. To address this, we reanalyzed the data excluding this subject, and found that all subsequent results still held, unless otherwise specified.

### 2.4 EEG recording and preprocessing

EEG data were collected using a 64-electrode cap (BioSemi MP150 System) with active electrodes and additional reference and ground electrodes. Electrooculograms were recorded via electrodes placed on the supraorbital and infraorbital ridges of the left eye and on the outer canthi of both eyes. Signals were digitised at 1024 Hz with a DC amplifier. EEG data were pre-processed and analyzed using SPM12 (http://www.fil.ion.ucl.ac.uk/spm/; version 7487) and Matlab (R2023a; version 9.14.0.2206163). Continuous EEG data were high-pass filtered (0.1 Hz), down-sampled to 256 Hz, and low-pass filtered (30 Hz) to ensure comparability with previous results (Weber et al., 2022). Data were epoched into 600 ms segments around tone onsets (-100 to 500 ms), and baseline correction was performed using a -100 to 0 ms peristimulus window. Eye movement artifacts were corrected using the signal space projection (SSP) eyeblink correction method in SPM12 (Nolte & Hämäläinen, 2001), which regresses out the leading component from the EEG data based on eye activity (seeSupplementary Materialfor further details). Following eyeblink correction, trials with amplitudes±75μVwere considered as artefactual and excluded. Channels with over 20% artefactual trials were interpolated for analyses. One participant had a single bad channel (P8). The median number of artifact-free trials, taken across all participants, was 1750 (25th percentile: 1623, 75th percentile: 1783). This total includes all recorded trials, encompassing not only trials defined as standard and deviant tones according to our definition but also all other trials presented during the task. Notably, our parametric model-based analysis incorporates data from all trials collectively, without differentiating between these conditions.

### 2.5 Difference waveform analysis

In-line withWeber et al., 2022, trial types were defined as follows: deviant trials followed at least 5 repetitions of the other tone (N = 119), and standard trials were defined as the 6th repetition of the same tone (N = 106). In the standard condition, the median number of artifact-free trials was 103 (25th percentile: 97, 75th percentile: 106), and in the deviant condition, it was 116 (25th percentile: 112, 75th percentile: 118). The difference waveform was obtained by subtracting the average standard ERP from the average deviant ERP. Additionally, separate stable and volatile difference waveforms were obtained by subtracting the standard ERP from the deviant ERP during the respective phases. In the stable phase, there were 51 standard tones and 55 deviant tones, whereas in the volatile phase, there were 55 standard tones and 64 deviant tones.

For each subject, difference waveform ERPs were converted into scalp images for all 64 channels using a voxel size of 4.3 mm x 5.4 mm x 2.0 ms. In agreement with previous studies (0, 0, 0, 0), statistical analyses were limited to a 100–400 ms post-stimulus interval, to capture the MMN and*P*300 peaks and to reduce the amount of comparisons. Images were smoothed with a Gaussian kernel (FWHM: 16 x 16 mm) to meet the assumptions of Gaussian random field theory (Kiebel & Friston, 2004;Worsley et al., 1996).

Images from the first-level served as input to our second-level general linear models (GLMs). We tested for the effect of difference waveform expression across sensor space and peristimulus time. Significant effects were inferred from thresholded*F*-statistical parametric maps (SPMs) at peak (*p*< 0.05) and cluster (*p*< 0.05) level, which were family-wise error corrected using Gaussian random field theory with a cluster-defining threshold of*p*< 0.001 (Flandin & Friston, 2019).

### 2.6 Computational framework

Single-trial EEG data were modeled using the HGF, a hierarchical Bayesian model of learning under uncertainty (Mathys et al., 2011,^2014^), which was previously used to model oddball paradigms (Charlton et al., 2022;Hauke et al., 2023;Weber et al., 2020) (Fig. 1B). The HGF was implemented using the*tapas_ehgf_binary*function of the HGF toolbox (version: 6.0), which is part of the open-source TAPAS software collection (version: 4.0.0) (https://github.com/translationalneuromodeling/tapas/releases/tag/v4.0.0) (Frässle et al., 2021).

Participants were exposed to a sequence of tones in which the high tone were either “deviant” or “standard” depending on their local frequency compared to low tones. We used the HGF to model an individual’s implicit learning of the tone sequence. Since the oddball paradigm does not require behavioral responses, and therefore the model cannot be fit to behavior, participants were modeled as surprise-minimizing Bayesian observers (seeSupplementary Materialfor more details). Perceptual parameters were optimized to reduce overall surprise in response to the experienced tone sequence (seeSupplementary Tables S1 and S2for a summary of the parameters).

According to the model, participants’ beliefs are recursively updated using the following equation:

$$\Delta \mu_{i}^{\left(k\right)}\propto \frac{{\hat{\pi }}_{i-1}^{\left(k\right)}}{\pi_{i}^{\left(k\right)}}\delta_{i-1}^{\left(k\right)}$$

where$\Delta \mu_{i}^{\left(k\right)}$denotes the change in the posterior belief at level*i*on trial*k*. After each tone is experienced, the prediction error from the level below ($\delta_{i-1}^{\left(k\right)}$) is computed and then weighted by a ratio of precisions: the precision of the prediction about the level below (sensory precision) (${\hat{\pi }}_{i-1}^{\left(k\right)}$) and the belief precision at the current level ($\pi_{i}^{\left(k\right)}$). The precision ratio functions as a dynamic learning rate, as it adjusts the magnitude of belief updates based on one’s confidence in the sensory input relative to their prior belief. For example, if the environment is stable, the learning rate may be small, resulting in more modest belief updates. By contrast, when the environment is volatile, the learning rate can be increased, enabling greater belief updates and providing a mechanism for flexible adaptation to changing environments.

From the model, we extracted trial-wise estimates of precision-weighted prediction errors (pwPE) at two levels of the hierarchy: low-level sensory pwPEs about the tone tendency$\varepsilon_{2}^{\left(k\right)}$and high-level volatility pwPEs that update the estimate of environmental volatility$\varepsilon_{3}^{\left(k\right)}$.

### 2.7 Sensor-level single-trial EEG analysis

EEG waveforms were converted to images and smoothed as outlined above. At the single subject level, a GLM with an intercept term and z-standardized computational trajectories was constructed to explain changes in EEG amplitude across trials. Given that precisions may be connected to neurotransmitter-NMDAR (N-methyl-D-aspartate receptor) interactions and PEs to AMPA (α-amino-3-hydroxy-5-methyl-4-isoxazolepropionic acid) signaling (Friston et al., 2016;Sterzer et al., 2018), two GLMs were included to unpack the effects of pwPEs. They consisted of the low-level unweighted PE$\delta_{1}^{\left(k\right)}$and the corresponding precision ratio ($\psi_{2}$), as well as the high-level unweighted PE$\delta_{2}^{\left(k\right)}$and corresponding precision ratio ($\psi_{3}$). The average collinearity between these regressors was r = 0.088±0.005 (mean±SD) and r = 0.119±0.004, respectively. Low-level pwPEs$\varepsilon_{2}^{\left(k\right)}$and high-level pwPEs$\varepsilon_{3}^{\left(k\right)}$also served as multiple regressors in an additional first-level GLM (*r*= 0.535±0.004). Consistent with previous studies (Weber et al., 2022), these regressors were not orthogonalized with respect to each other.

The same model-trajectories were used for all participants; however, following EEG preprocessing, these trajectories varied slightly due to rejected trials following artifact rejection. For each computational quantity, we conducted an*F*-test at each point in 2D sensor space and each time point to test the null hypothesis that the correlation between the model-derived learning trajectory and EEG amplitudes was zero, generating beta images that were used for second-level analysis.

At the group level, we specify separate GLMs for each computational quantity, in which beta images from the first-level served as input to the second-level. We identified significant effects using the same threshold criteria as described inSection 2.5.

### 2.8 Source-level EEG analysis

Finally, we applied multiple sparse priors (MSP) source reconstruction to estimate the distribution of cortical sources that give rise to the sensor level single-trial EEG data (Friston et al., 2008). We defined a prior set of sources in line with previous MMN studies, indicating the involvement of specific cortical regions, including bilateral primary auditory cortices (A1), bilateral superior temporal gyri (STG), and bilateral inferior frontal gyri (IFG) (Garrido et al., 2008,Garrido, Kilner, Kiebel, et al., 2009;Giard et al., 1990;Molholm et al., 2005;Opitz et al., 2002) (seeSupplementary Table S3for source coordinates). Source reconstruction was applied to both MMN waveforms (MMN analysis) and single-trial data (computational analysis) to estimate source time courses. We further compared MSP with identically distributed (IID) source reconstruction, finding evidence in favor of MSP, and thus proceeded with the MSP analysis for all subsequent analyses (seeSupplementary Materialfor details). For the computational analysis, we proceeded as in the sensor space analysis and defined three design matrices GLMs (low-level pwPE design:$\delta_{1}$and$\psi_{2}$; high-level pwPE design:$\delta_{2}$and$\psi_{3}$; pwPEs design:$\varepsilon_{2}$and$\varepsilon_{3}$) with an intercept term and z-standardized computational trajectories to explain changes in absolute source amplitude across trials at the first level. First-level betas (reflecting the correlation between absolute source amplitude and computational quantity) were converted to images and carried to the second-level to test in which of the six sources and when in time each computational variable was expressed. The same statistical thresholds as in the sensor analysis were used.

## 3 Results

### 3.1 Effect of stability on mismatch negativity

**Fig. 2.**
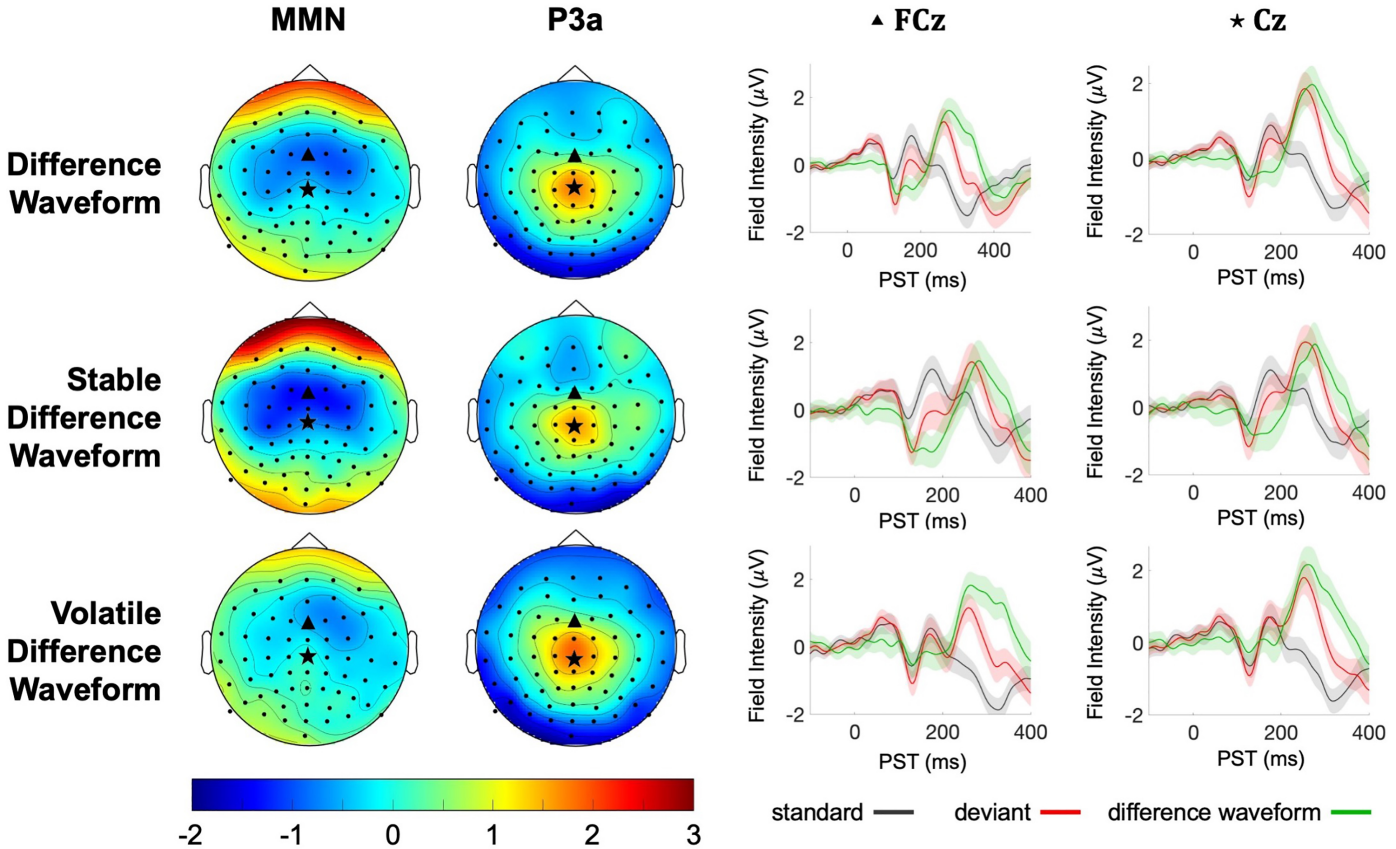
Event-Related Potential (ERP) Scalp Topographies: On the left, scalp voltage topography maps displaying group mean grand-average ERPs. The MMN component was averaged from 150 to 200 ms post-stimulus, and the P3a component was averaged from 250 to 300 ms post-stimulus. All maps are plotted on the same voltage scale (μV). On the right, event-related potential difference waveforms at the FCz and Cz electrodes are shown for the overall difference waveform for the entire paradigm, along with the stable and volatile difference waveforms.

Our sensor-level EEG analysis examined the influence of volatility on the mismatch response by comparing the difference waveform (deviant–standard) between stable and volatile periods (Fig. 2). For the stable condition,*F*-tests revealed a significant peak at 141 ms in frontal electrodes (peak,*F*_(1,42)_= 113.94;*p*< 0.001), aligning with the timing of the MMN effect. In the volatile condition, a significant peak occurred at 145 ms in frontal electrodes (peak,*F*_(1,42)_= 25.70;*p*= 0.034) (Supplementary Fig. S1).

**Fig. 3.**
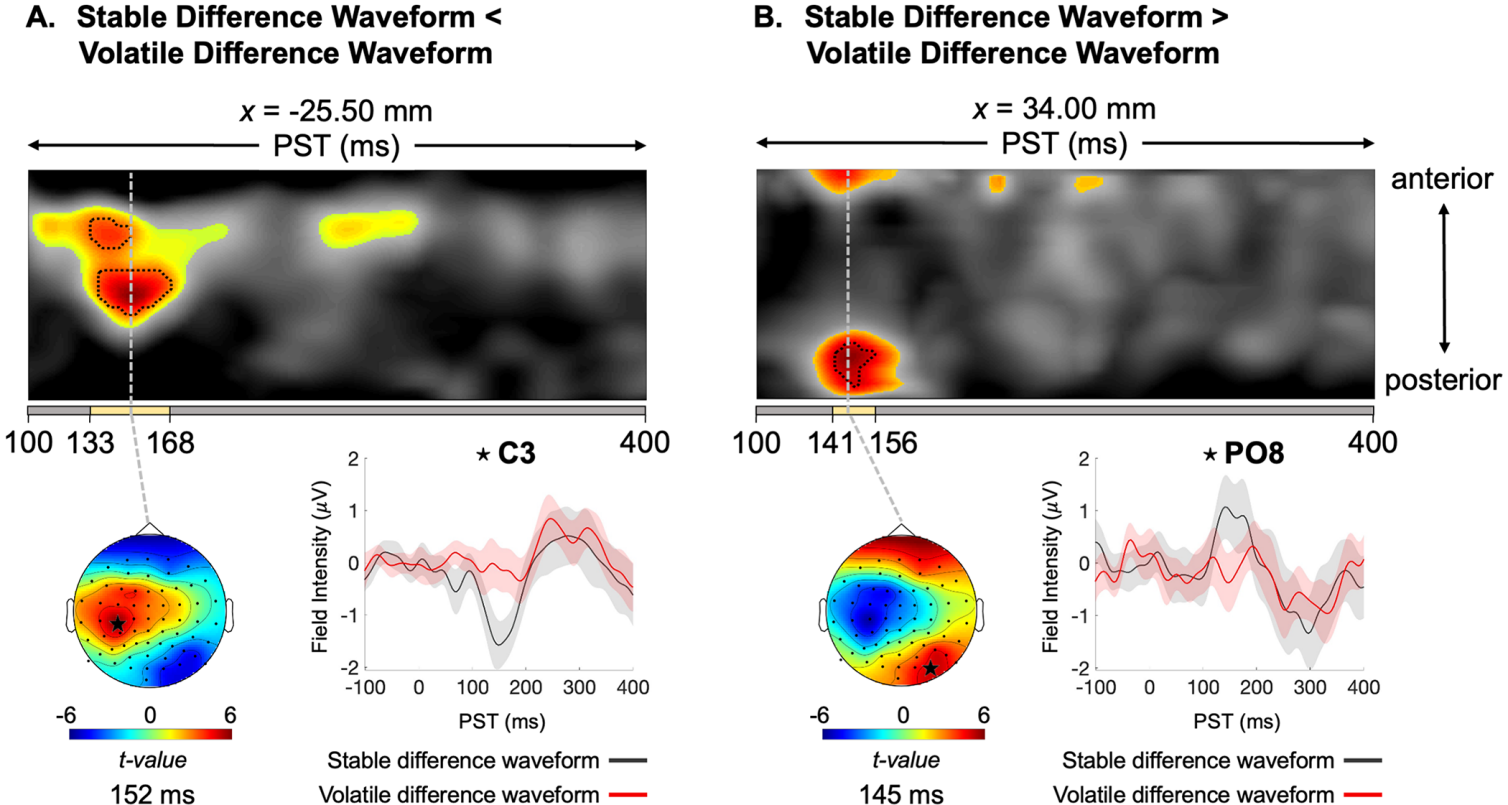
Mismatch responses in volatile phases are reduced compared to stable phases. (A) Maximum intensity projection*t*-map illustrating the contrast between stable MMN < volatile MMN ERPs across anterior to posterior scalp locations (top). Significant peak-level effects (*p *<0.05, whole-volume FWE-corrected) are outlined by black contours, while the coloured area indicates*t*-values exceeding the cluster-defining threshold of*p *<0.001, uncorrected. The yellow bar at the bottom of the*t*-map indicates the time range of significant peak effects, from earliest to latest significant time points. On the left, the scalp map displays the peak effect of the given cluster using an*F*-map at the indicated PST, displayed on a 2D sensor layout. On the right, difference waveforms (deviant–standard) are shown for each phase, with the chosen sensor location on the scalp map marked with a star. (B) Expression of clusters in time x sensor space where ERPs to tones in stable phases were more positive than ERPs to tones in volatile phase. MMN: mismatch negativity; PST: peri-stimulus time; FWE: family wise error.

Comparing stable versus volatile phases revealed significant differences in the waveform amplitude. The difference waveform was significantly larger in stable phases peaking at 152 ms in central electrodes (peak,*t_(1,42)_*= 5.9;*p*= 0.002;Fig. 3A) and 145 ms in parietal-occipital electrodes (peak,*t_(1,42)_*= 5.1;*p *=0.019;Fig. 3B). The difference wave showed greater negativity during stable phases in central electrodes, while parietal clusters exhibited the opposite pattern due to polarity changes. The left-lateralized effect observed in central electrodes (Fig. 3A) likely results from a relatively weaker MMN component on the left side in the volatile condition, resulting in a more pronounced stable-volatile difference in the waveforms.

### 3.2 Relationship between mismatch stability and functional impairment

**Fig. 4.**
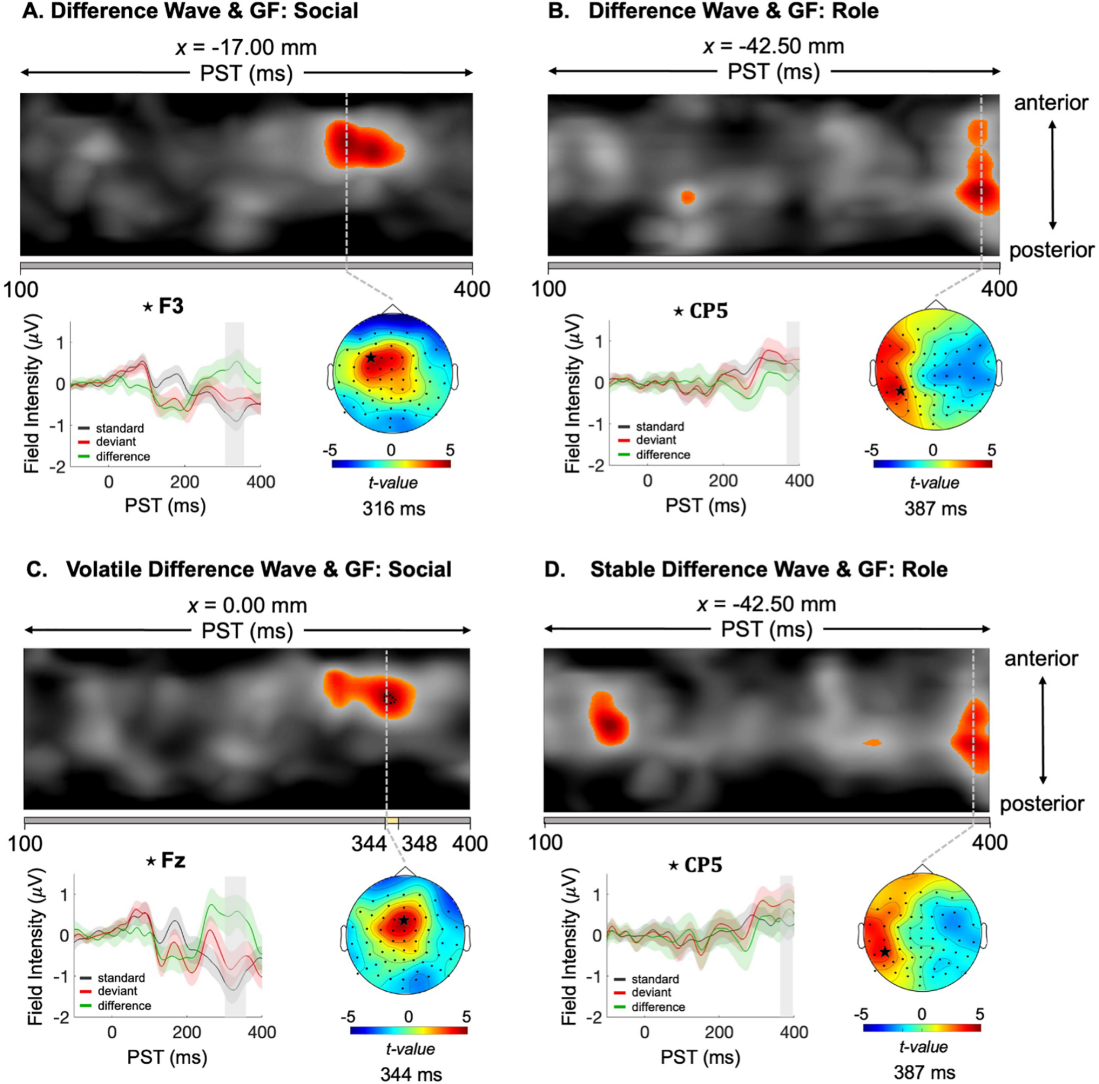
Positive correlation of the difference waveform with psychosocial function. (A–D) Maximum intensity projection*t*-map illustrating the correlation between global function (GF) and difference waveform ERPs across and anterior to posterior scalp locations (top). Significant peak-level effects (*p *<0.05, whole-volume FWE-corrected) are outlined by black contours, while the colored area indicates*t*-values exceeding the cluster-defining threshold of*p *<0.001, uncorrected. The yellow bar at the bottom of the*t*-map indicates the time range of significant peak effects, from earliest to latest significant time points. In the absence of a yellow bar, only cluster-level effects were significant. Note the statistical analysis was limited to 100–400 ms post-stimulus. On the left, the grand-average ERPs for standard, deviant, and their difference waveform (deviant – standard) at the peak effect location are shown. The shaded gray area indicates the duration of the significant effect for the specified cluster. On the right, the scalp map highlights the selected sensor location, marked by a star. The scalp map displays the peak effect of the given cluster using an*F*-map at the indicated PST, displayed on a 2D sensor layout. PST: peri-stimulus time; FWE: family wise error.

Next, we examined the relationship between the sensor-level difference waveform across both phases (stable and volatile) and functioning at baseline. Significant positive correlations were found between the difference waveform and GF: Social, peaking at 332 ms in central-frontal electrodes (cluster,*t_(1,41)_*= 4.5;*p*= 0.003;Fig. 4A), and GF: Role, peaking at 387 ms in temporal electrodes (peak,*t_(1,41)_*= 4.7;*p*= 0.049;Fig. 4B).

We investigated the relationship between functional impairment and stability, revealing a significant positive effect between the volatile difference waveform and GF: Social, peaking at 344 ms in central-frontal electrodes (peak,*t_(1,41)_*= 4.8;*p*= 0.036;Fig. 4C). We also observed a significant positive correlation between the stable difference waveform and GF: Role, peaking at 387 ms in temporal electrodes (cluster,*t_(1,41)_*= 4.3;*p*= 0.049;Fig. 4D). However, after excluding one subject with poor behavioral performance (based on a hit-rate below 75% in the visual distraction task), the effect with GF: Role fell just short of significance, possibly due to a loss of statistical power (cluster,*t_(1,40)_*= 4.3;*p*= 0.054).

These effects remained significant when including either working memory performance (assessed with the digit span backward task (Wechsler, 1981)), age, or cannabis consumption as covariates, except for the correlation between GF: Role and stable difference waveform (Fig. 4D). This correlation was only marginally significant when either working memory, age, or cannabis was included as a covariate (*p_cluster_*= 0.063,*p_cluster_*= 0.054 and*p_cluster_*= 0.087 respectively). Given the borderline significance of this result prior to covariate adjustment (*p_cluster_*= 0.049), this result should be interpreted with caution and further validation with a larger sample size is needed. Notably, none of these covariates had a significant impact on the difference waveform.

### 3.3 Cortical source of mismatch stability and functional impairment relationship

**Fig. 5.**
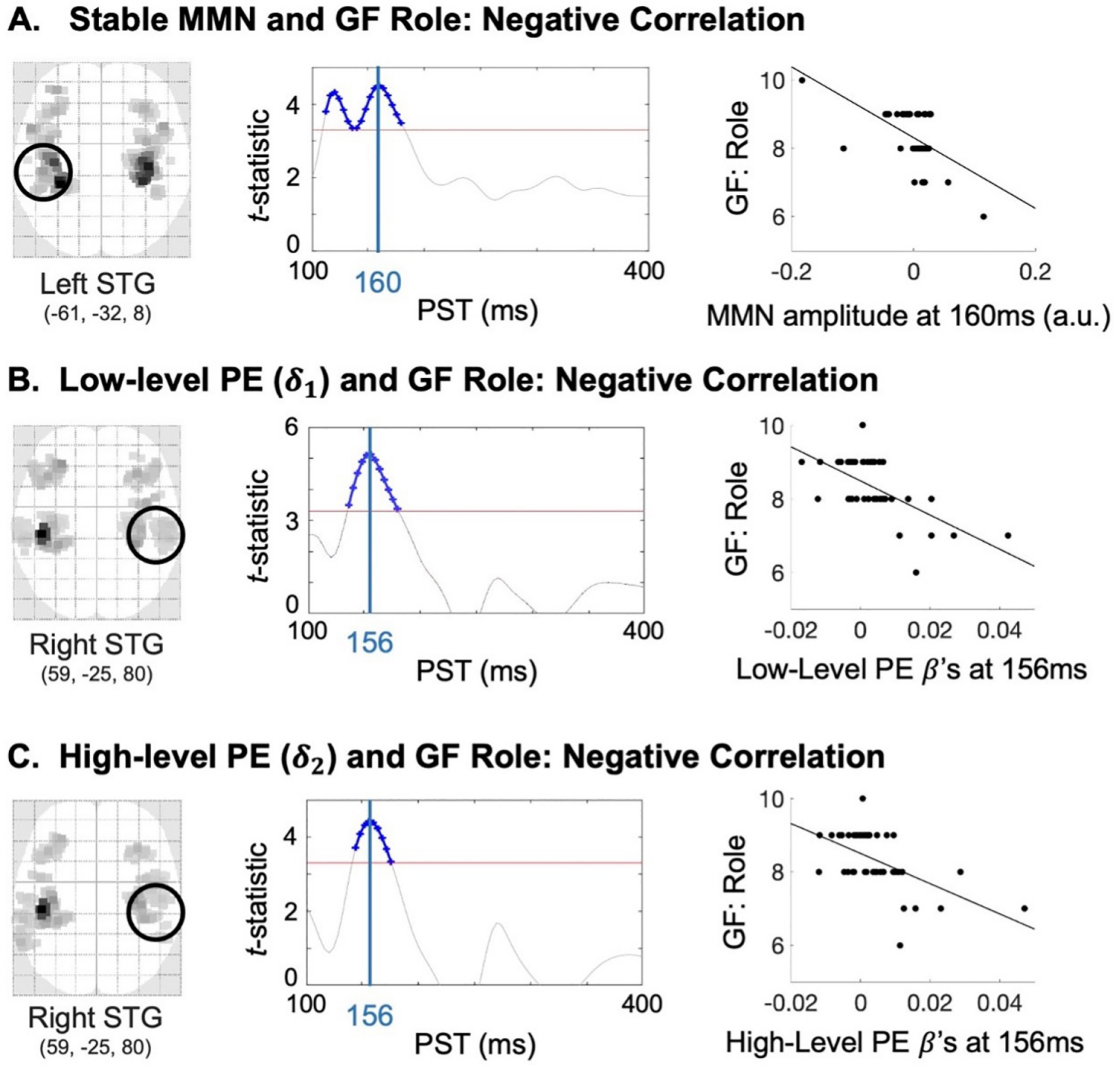
Cortical sources associated with functional impairment. (A) A negative correlation between the stable mismatch response and GF: Role scores were observed, peaking at 160 ms (301–309 ms) in the left superior temporal gyrus (STG). The source activation estimated from the difference waveform (MMN) at the peak time point (160 ms) is depicted using an SPM-glass brain (left). Significant*t*-contrasts of the stable mismatch response and global function (GF) scores are presented for the given source (left STG) over peri-stimulus time (PST) (middle). The significance threshold using peak-family wise error (FWE) correction is indicated by the red horizontal line, and all time points above this threshold are colored in blue. The peak time point is marked by the blue vertical line. The scatter plot (right) illustrates the relationship between GF scores and the stable MMN amplitude at the peak time point (160 ms) and source (left STG). (B) A negative correlation between the low-level ($\delta_{1}$) and (C) high-level ($\delta_{2}$) unweighted prediction errors (PEs) and global function (GF): Role scores was observed, peaking at 156 ms in the right STG. The source activation was estimated from the grand-averaged difference waveform (10% highest–10% lowest$\delta_{1}$or$\delta_{2}$trials, respectively) at the peak time point (156 ms). MMN: mismatch negativity; PST: peri-stimulus time; GF: global function; A1: primary auditory cortex; STG: superior temporal gyrus.

Next, we identified the cortical sources underlying the difference waveform and its correlations with functioning, as shown inFigure 4. Source activity of the difference waveform is detailed in theSupplementary Material. We found a significant negative correlations between GF: Role and source activity in left STG peaking at 160 ms for the stable difference waveform (peak,*t_(1,41)_*= 4.8;*p*< 0.001;Fig. 5A). Notably, this effect in source space coincides with the cluster identified in our sensor space analysis, which exhibited marginal significance in sensor space, peaking at 145 ms in the left temporal region (peak,*t_(1,41)_*= 4.1;*p*= 0.056;Fig. 4D). The presence of a significant effect in source space might suggest that our sensor space analysis lacked sufficient statistical power, as the sensor analysis was conducted across the entire sensor space, whereas the source analysis was constrained to six sources that were previously associated with MMN generation.

### 3.4 Expression of hierarchical prediction errors and precision ratios

**Fig. 6.**
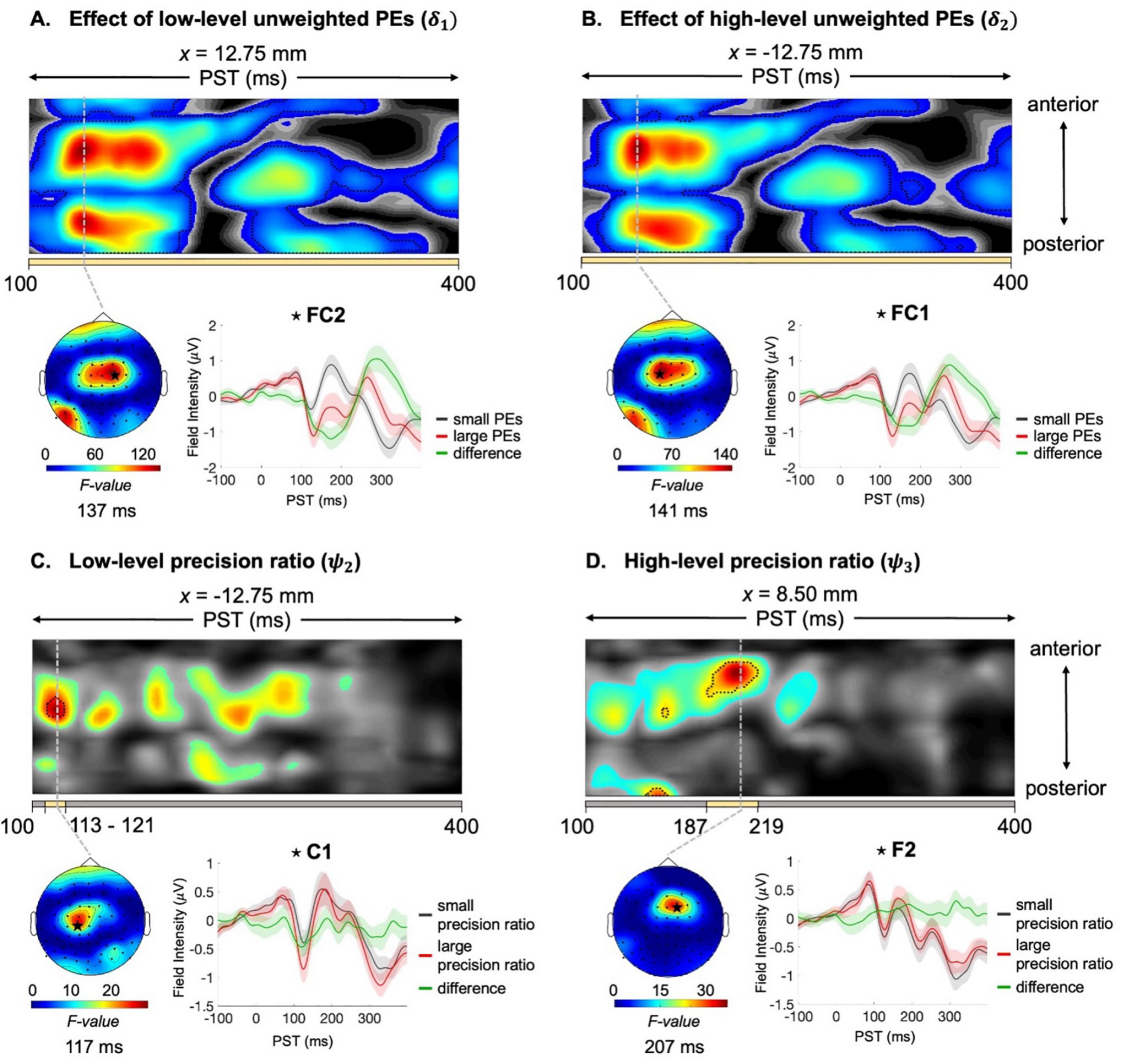
Unweighted prediction error (PE) and precision ratio expression. Maximum intensity projection*F*-map illustrating the effect of (A) low-level sensory PEs ($\varepsilon_{2}$), (B) high-level volatility PEs ($\varepsilon_{3}$), (C) low-level sensory precision ratio ($\psi_{2}$), and (D) high-level volatility precision ratio ($\psi_{3}$) on EEG amplitudes across anterior to posterior scalp locations (top). Significant peak-level effects (*p *<0.05, whole-volume FWE-corrected) are outlined by black contours, while the colored area indicates*F*-values exceeding the cluster-defining threshold of*p *<0.001, uncorrected. The yellow bar at the bottom of the*F*-map indicates the time range of significant peak effects, from earliest to latest significant time points. On the left, the scalp map displays the peak effect of the given cluster using an*F*-map at the indicated PST, displayed on a 2D sensor layout. On the right, ERPs were averaged across the electrode at the peak of the significant clusters using the 10% largest and 10% smallest PE (or precision ratio) values. The selected electrode position indicated by a star on the scalp map. MMN: mismatch negativity; PST: peri-stimulus time; FWE: family wise error.

At the sensor level, we observed significant correlations between the low-level (sensory) and high-level (volatility) unweighted PEs and precision ratio trajectories with EEG amplitude (Fig. 6). Specifically, low-level PEs showed an early peak at 137 ms in frontal-central electrodes (peak,*F*_(1,42)_= 140.9;*p*< 0.001;Fig. 6A), while high-level PEs peaked at 141 ms in frontal-central electrodes (peak,*F*_(1,42)_= 136.2;*p*< 0.001;Fig. 6B). Additionally, the low-level precision ratio peaked at 117 ms in central electrodes (peak,*F*_(1,42)_= 28.9;*p*= 0.015;Fig. 6C) whereas the high-level precision ratio peaked at 207 ms in frontal electrodes (peak,*F*_(1,42)_= 36.3;*p*= 0.002;Fig. 6D). Correlations were also found between trial-wise EEG activity and both low-level and high-level pwPEs (Supplementary Fig. S2). No significant correlations were found between the model trajectories and functioning in sensor space.

### 3.5 Cortical sources of hierarchical prediction errors and functional impairment relationships

Next, we analyzed the cortical generators of these sensor-level unweighted PE and precision effects. Our findings revealed low-level and high-level unweighted PE expression in the left STG (peak,*F*_(1,42)_= 17.4;*p*= 0.003 and peak,*F*_(1,42)_= 21.2;*p*= 0.001, respectively), as well as high-level unweighted PE expression in the left (peak,*F*_(1,42)_= 18.3;*p*= 0.003) and right A1 (peak,*F*_(1,42)_= 22.4;*p*= 0.001).The left A1 was additionally associated with high-level precision ratio (peak,*F*_(1,42)_= 12.8;*p*= 0.022).

Although no correlations with function were found at the sensor level, significant correlations emerged at the source level. Similar to our mismatch analysis, this difference could be attributed to the increased power in the source analysis. Notably, significant negative correlations emerged between GF: Role and the expression of both low-level unweighted PE (peak,*t*_(1,41)_= 5.2;*p*< 0.001;Fig. 5B) and high-level unweighted PE (peak,*t*_(1,41)_= 4.4;*p*= 0.001;Fig. 5C) in the right STG peaking at 156 ms. These source effects remained significant when including either working memory performance, age, or cannabis consumption as covariates (seeSupplementary Materialfor detailed discussion on the effect of covariates in the source-space analysis).

## 4 Discussion

In this study, we investigated the EEG signatures of hierarchical predictive errors during ERP generation using a modified auditory oddball paradigm in healthy controls. We found that stability significantly impacted the difference waveform, with a reduced expression of the auditory MMN in volatile compared to stable phases. Importantly, despite using a novel auditory oddball task, these findings replicate established effects in the literature (Dzafic et al., 2020;Kotchoubey, 2014;Todd et al., 2011,^2014^;Weber et al., 2022), confirming the validity and effectiveness of our experimental approach.

Importantly, we found that this difference waveform was differentially related to psychosocial functioning, with the stable condition correlating with Global Functioning: Social scores and the volatile condition with Global Functioning: Role scores. Additionally, we observed that both PEs and precisions were expressed during the oddball task, with PEs correlating with ERPs over a larger time window and precision ratios at distinct points, and these PE expressions were linked to functioning in the superior temporal gyrus. These findings highlight the nuanced role of predictive coding in social and role functioning, offering insights into the neurobiological underpinnings of psychosocial behaviors.

### 4.1 Difference waveform and psychosocial functioning

We observed a positive correlation between the difference waveform across the entire paradigm and GF: Social scores, peaking at 332 ms post-stimulus (Fig. 4A). This correlation was significant during the volatile phase of the paradigm, peaking between 344 and 348 ms post-stimulus (Fig. 4C), but was not significant during the stable phase. The timing and topography of this correlation corresponded with the P3a component, suggesting that smaller P3a amplitudes may be associated with reduced psychosocial functioning in HCs (Light et al., 2007), a finding replicated across psychosis spectrum disorders (Carrión et al., 2015;Hermens et al., 2010;Murphy et al., 2020). Given the age-related decline of P3a amplitudes (Kiang et al., 2009) and its reduction across clinical populations (Chen et al., 2015;Kaur et al., 2011;Kim et al., 2020;Light et al., 2015;Solís-Vivanco et al., 2015), a blunted P3a response may serve as an indicator of broader cognitive dysfunction.

This is particularly relevant given the inherent volatility of the social world, where accurate interpretation of social cues—integrating facial expressions, body language, tone of voice and conversational context—is crucial for inferring others’ intentions. Moreover, these cues must be weighed against their relative reliability (Griffin & Fletcher, 2017) and a failure to accurately integrate and interpret social information may lead to the generation of aberrant beliefs about others’ intentions or actions (Diaconescu et al., 2019).

Moreover, we identified an additional correlation between the difference waveform during the stable phase and GF: Role scores (Fig. 4B, D). Although this effect was of marginal significance, it suggests a potential link between neural responses and role functioning even in stable conditions. Caution is warranted in interpreting this finding, and replication in a larger sample is required to substantiate this effect.

Our source-level analysis further revealed that a more positive stable difference waveform amplitude within the left STG, peaking at 160 ms post-stimulus, correlated with poorer role functioning. This effect coincides with the timing of the MMN component. While previous studies have localized the MMN to the left STG and observed reductions in MMN amplitude among patients with schizophrenia (Erickson et al., 2016), our study is the first to link MMN amplitude in the left STG to role functioning within a healthy population. Interestingly, the left STG has consistently shown grey matter loss in patients with schizophrenia (Vita et al., 2012), and reductions in MMN amplitude have been associated with poorer psychosocial functioning in this population (Hamilton et al., 2018;Thomas et al., 2017). Our findings suggest that even among healthy individuals, variability in MMN amplitude in the left STG may be linked to individual differences in role functioning, offering new insights into the neurobiological mechanisms underlying psychosocial behaviors.

### 4.2 Multiple, hierarchically-related prediction errors underlie the MMN

In line withWeber et al. (2022)and other single-trial MMN analyses (Hauke et al., 2023;Stefanics et al., 2018;Weber et al., 2020), we found significant associations between hierarchical PE expression and trial-wise EEG activity (Fig. 6). Notably, correlations between unweighted PEs and EEG amplitudes occurred mainly in three distinct time windows or cluster groups, potentially corresponding to the MMN, P3a, and reorienting negativity (RON) component. The RON, characterized by a negative waveform, signals attentional reorientation, expressed after the P3a component in frontal electrodes. These findings suggest a potential link between the MMN/P3a/RON complex and hierarchical PE expression.

Previous studies have investigated hierarchical PE expression in the context of MMN generation using oddball-like paradigms, such as the local-global paradigm. However, the nature of PEs identified through the HGF is distinct from those identified in the local-global paradigm. In the HGF, hierarchical levels are coupled via their variances, implying a temporal hierarchy where higher levels reflect the slower rates of change relative to the level below (Hauke, 2022). In contrast, the local-global paradigm assumes a hierarchy based on “what” is changing—that is, local or global deviance. Hence, the use of hierarchical Bayesian models is useful for understanding the dynamics of change, rather than just the occurrence of change.

Using source reconstruction, we found low-level and high-level unweighted PE expression in the left STG, coinciding with the timing of the MMN, and high-level precision ratio expression in the right STG, partially aligning with the P3a component. Contrary to our initial hypothesis that low-level PEs would be generated in early sensory regions and high-level PEs in higher-level regions, our findings suggest a more complex distribution of predictive coding processes across brain regions. Additionally, a negative correlation between GF: Role and unweighted low- and high-level PEs in the right STG was found at 156 ms post-stimulus, highlighting the STG’s involvement in PE computation, with increased PE expression linked to diminished role functioning. Notably, this effect aligns with the correlation between the positive stable difference waveform amplitude and role functioning within the left STG, peaking at 160 ms post-stimulus. Taken together, these ERP-based and model-based findings suggest a role for the STG in MMN–PE expression and its association with role functioning.

Furthermore, higher volatility precision ratios (i.e., learning rate) correlated with decreased social function in the primary auditory cortex at 254 ms, suggesting that individuals with lower social function may be more sensitive to environmental volatility. Previous work has shown that increased expectations of volatility are associated with higher levels of paranoia (Reed et al., 2020) and emerging psychosis (Cole et al., 2020;Hauke et al., 2024). This finding aligns with our earlier observations where the volatile difference waveform correlated with GF: Social (Fig. 4C), indicating a potential role for volatility precision ratio in later P3a expression. Higher cognitive levels (e.g., frontal cortex) may represent expected predictions (e.g., anticipated tone), while lower levels (e.g., primary auditory cortex) compute weighted PEs about the tones and environmental volatility.

Notably, despite using a non-social task with healthy controls, we still observed that changes in volatile MMN expression and volatility learning were associated with reduced social functioning, specifically. More investigation is needed to understand how social context influences volatility perception in both healthy individuals and across psychosis spectrum disorders (Gibbs-Dean et al., 2023).

### 4.3 Limitations

This study has several limitations. First, as the MMN paradigm is a passive task, the HGF model could not be fit to participants’ behavior. Instead, belief trajectories were simulated based on the assumption of a Bayes-optimal learner, consistent with previous studies (Hauke et al., 2023;Weber et al., 2020,^2022^). Future research should consider employing an active oddball task (Hamilton et al., 2019), which would allow for direct measurement of participants’ behavior. This approach would allow us to perform model selection based on participants’ behavior and to test the current computational approach against other theoretical models of MMN generation (Lieder, Stephan, et al., 2013). Second, since the data presented here were collected as part of an early psychosis study (Hauke et al., 2024), the functioning assessment employed was not tailored to or developed for the assessment of broader populations (Cornblatt et al., 2007). However, our results suggest that there are relevant relationships between early auditory information processing and psychosocial functioning. Thus, future studies should include more comprehensive assessments of functioning to better characterize the effects (e.g., WHDAS 2.0) (Gold, 2014).

### 4.4 Future directions

Future research should leverage computational models with greater physiological detail, like connectome-based neurophysiological modeling (Momi et al., 2023) and dynamic causal modeling (David et al., 2006;Friston et al., 2003,^2019^), to delve deeper into the neurobiology of hierarchical PEs and MMN expression. These models can shed light on local neural dynamics, receptor densities, and the interplay between precisions, PEs, and AMPAR/NMDAR function. Finally, to enhance the external validity of our findings related to early information processing in HCs, we recommend that future studies incorporate larger sample sizes and measures like WHODAS 2.0 to bolster the generalizability of the results.

## 5 Conclusion

In conclusion, our exploratory study investigated the neural correlates of hierarchical PEs during MMN generation using a novel auditory oddball paradigm. We found a significant effect of stability on the mismatch difference waveform, with larger responses in stable phases and associations with psychosocial functioning. Moreover, we find evidence for the role of hierarchically-related PEs in the generation of the MMN and their association with functioning in the superior temporal gyrus. These results underscore the importance of predictive coding in understanding in early auditory information processing and psychosocial functioning.

## Supplementary Material

Supplementary Material

## Data Availability

The analysis code for this study is publicly available athttps://github.com/colleenc11/COMPI_MMN. The data is publicly available athttps://osf.io/exsj3/.
