# Supplementary material for: Localizing hierarchical prediction errors and precisions during an oddball task with volatility: Computational insights and relationship with psychosocial functioning in healthy individuals
Source: Imaging Neurosci (Camb). 2025 Feb 3;3:imag_a_00461. doi: 10.1162/imag_a_00461 (PMC12319832; doi:10.1162/imag_a_00461)
Supplement: Supplementary Material [file imag_a_00461-supp.pdf]

Supplementary

Inclusion and Exclusion Criteria

Participants were included if they were above 14 years of age. Exclusion criteria included the following: previous psychotic episodes, psychotic symptomatology secondary to an organic disorder, any neurological disorder (past of present), premorbid IQ < 70 (as determined by the digit span backwards task from the Wechsler Adult Intelligence Scale-Revised (Wechsler, 1981)), color-blindness, substance use disorders according to ICD-10 criteria (except cannabis), consumption of alcohol or cannabis within 24 hours prior to measurements, and regular drug consumption (except alcohol, nicotine, and cannabis). The latter was verified with a drug screening before the EEG measurement. If a positive result was found, assessments were delayed until a negative result was obtained.

Behavioral Analysis

During auditory stimuli presentation, participants performed a visual distraction task that required them to detect changes of a small white square displayed at the center of the screen. This task consisted of 36 'square openings' occurring at uneven intervals and not overlapping with tone presentations, with a random delay of 50-250 ms after the tone's conclusion.

We computed the mean reaction times and hit rates of participants based on their responses to the visual targets, where hit rates refer to the proportion of correct responses out of the total number of visual targets presented. On average, participants responded to visual targets after 484.6 ms (SD = 61.5) and correctly responded to 92.4% (SD = 11.8) of the presented targets.

Eyeblink Correction

Eyeblink events were detected via a thresholding approach applied to the vertical EOG channel data, with a default eyeblink detection threshold of 3 SD. The continuous EEG was segmented into 1000 ms segments (-500 ms to 500 ms) around detected eyeblink events. Signal space projection (SSP) was used for the detection and removal of eyeblink components. Ocular components were determined using singular value decomposition (SVD) of topographies from all eyeblink trials and all time points. The leading SVD component was used to define the noise subspace that was removed from the spatial subspace spanned by the eyeblink components in the continuous data (Nolte et al., 2001). Eyeblink detection thresholds were manually inspected and adjusted when necessary, with a lowered threshold of 2.5 and 2 SDs for two HC with shallow eyeblinks. Eyeblink correction was evaluated by visually inspecting average eye blink ERPs before and after correction. Based on this inspection and to ensure that the eyeblink correction was neither over- nor under correcting, eyeblink data was precleaned before computing ocular components for a subset of six participants. This involved lowpass filtering the data (10Hz cut-off) and rejecting epochs with large transients ($> \pm 500\ \mu V$). Furthermore, one additional eyeblink component was removed for five participants.

Computational Model

According to the model, an agent maps the sensory inputs u, which for a given trial k denotes a low ($u^{(k)} = 0$) or high tone ($u^{(k)} = 1$), to three hidden states. The inferred hidden states are organized as follows: the lowest level $x_1^{(k)}$ corresponds to the *tone probability* (i.e., probability of hearing a high tone). At the second level $x_2^{(k)}$, the probably for a high tone ($x_1^{(k)}$) is transformed into an

unbounded state $(-\infty, +\infty)$, referred to as *tone tendency*, while the third level $x_3^{(k)}$ captures the *environmental volatility*, referring to changes in the tone tendency over time.

Individual learning and belief trajectories are modulated by three participant-specific perceptual parameters, namely κ, ω_2, and ω_3. The coupling strength between the second and third levels is determined by κ, while ω_2 denotes the tonic component of the log-volatility at the second level that determines the rate of learning about tone tendency. On the other hand, ω_3 refers to the variability of volatility over time (meta-volatility) that controls the rate of learning about environmental volatility. The prior settings (mean, variance) of these parameters were chosen based on previous computational studies of the MMN (Charlton et al., 2022; Weber et al., 2020) and are summarized in Table S1. The parameters following model inversion are summarized in Table S2.

Source Reconstruction

To evaluate the robustness of our findings, we compared multiple sparse priors (MSP) source reconstruction with independent and identically distributed (IID) source reconstruction. The IID approach does not constrain the sources to predefined regions, thus allowing for a more flexible interpretation of source activity location. Using fixed-effects Bayesian model selection (Stephan et al., 2009), we tested whether IID provides a better explanation of the data compared to MSP. Our analysis revealed a log group Bayes factor (GBF) of 3.8233e+06, providing strong evidence (log GBF > 3; (Kass et al., 1995)) in favor of the MSP source reconstruction. This comparison supports the validity of our choice of six specific regions based on previous literature (Adams et al., 2022; Garrido et al., 2008; Garrido et al., 2009; Phillips et al., 2015), indicating that the MSP

method is a robust and reliable approach for modeling cortical sources in this context. The source coordinates for MSP source reconstruction are summarized in Table S3.

Mismatch negativity expression and association with global function

Table S4 presents the sensor-level summary statistics for the ERP difference waveform during stable and volatile periods. The table includes information on cluster size, whole-volume FWE-corrected p-values at the cluster and peak level, peak F-values, and peak cluster coordinates (x and y for space and z for time). Table S5 presents similar summary statistics, specifically the t-statistic for the comparison of the stable and volatile difference waveforms. Finally, Table S6 presents summary statistics for the associations between the ERP difference waveform and Global Function: Role and Social scores.

Furthermore, cortical sources of the difference waveform (deviant - standard) were examined through source-level analysis, encompassing the entire paradigm as well as within stable and volatile phases (Table S7). We found a significant effect of the difference waveform in the left A1, peaking at 246 ms post-stimulus (peak, $F_{(1,42)} = 21.3$, $p = 0.001$) and in the right A1 at 281 ms post-stimulus (peak, $F_{(1,42)} = 17.9$, $p = 0.002$). Additionally, there was a significant effect of the volatile difference waveform in the left A1 at 285 ms post-stimulus (peak, $F_{(1,42)} = 15.0$, $p = 0.007$). There was no significant source effect of the stable difference waveform. In Section 3.3 of the main text, source-level correlations with GF and the difference waveform are discussed and summary statistics are provided in Table S8.

Sensor-Level Model-Based Results

Significant sensor-level model-based effects are summarized using maximum intensity projections of significant clusters over left to right scalp locations, retaining the anterior-posterior and PST dimensions in the plots. Figure S2 shows EEG signatures of precision-weighted PEs (ε_2,ε_3). For low-level sensory precision-weighted PEs ε_2, an early group of clusters occurred in frontal electrodes between 117 and 223 ms (peak, $F_{(1,42)} = 42.7$; $p = 0.001$; Figure S2A). A second group of clusters occurred later in central electrodes, between 250 and 305 ms (peak, $F_{(1,42)} = 32.1$; $p = 0.006$; Figure S2A).

For high-level volatility precision-weighted PEs ε_3, we observed three significant cluster groups, with the first group of clusters occurring between 105 and 230 ms in frontal-central electrodes (peak, $F_{(1,42)} = 123.4$; $p<0.001$; Figure S2B), a second group between 246 and 344 ms in central electrodes (peak, $F_{(1,42)} = 57.8$; $p<0.001$; Figure S2B), and a later group between 371 and 400 ms in central electrodes (peak, $F_{(1,42)} = 37.7$; $p = 0.002$; Figure S2B). Notably, the overlap in pwPE expression may be influenced by their underlying components, specifically the unweighted PEs and precision ratios. Our findings suggest that unweighted PEs at different hierarchical levels overlap in time, indicating a parallel processing of errors, potentially facilitated by recursive messaging (Jardi & Denève, 2013). In contrast, precision computations occur at more distinct time points, suggesting a more sequential computation (Figure 6). Table S9 presents the summary statistics for the model-based single trial analyses and event-related potential difference waveform.

Source-Level Model Based Results

The source-level model-based analysis was perform following an identical approach as for the sensor-level analysis. First-level GLMs with computational variables as explanatory factors were

used to identify source waveform correlates. At the group-level, GLMs for each computational variable were used to explain observed event-related responses on a single-trial basis, over sources and PST. Table S10 provide the summary statistics for the source-level model-based analyses.

Additional GLMs were used for each computational variable, while considering GF as a covariate. Correlations with GF: Role are visualized in Figure 5 of the main text, and summary statistics are provided in Table S11.

Effect of Covariates in Source-Level Analysis

Finally, we tested for the effect of working memory performance, age, or cannabis consumption as covariates in our source-level analysis (Table S12). We found a significant correlation between age and source activity for the volatile MMN in the right A1 at peaks of 160 ms, 199 ms, and 320 ms, in the left STG peaking at 320 ms, and in the right STG peaking at 344 ms. Additionally, we found significant correlations between age and source activity in the left IFG, peaking at 102 ms for the low-level precision ratio and at 297 ms for the high-level precision ratio. There was also a correlation between working memory and right STG activity for the difference waveform, peaking at 312 ms. It is worth noting that the cluster size for the majority of these associations is less than two voxels, indicating a relatively weak and small relationship.

Supplementary Figures

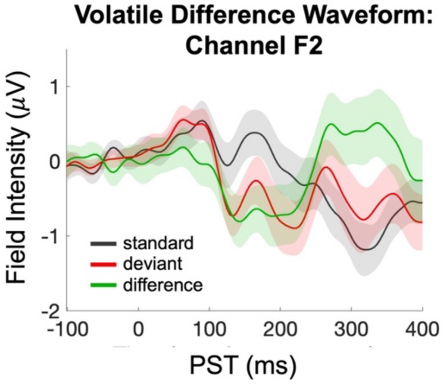

Figure S1: Volatile difference waveform at channel F2.

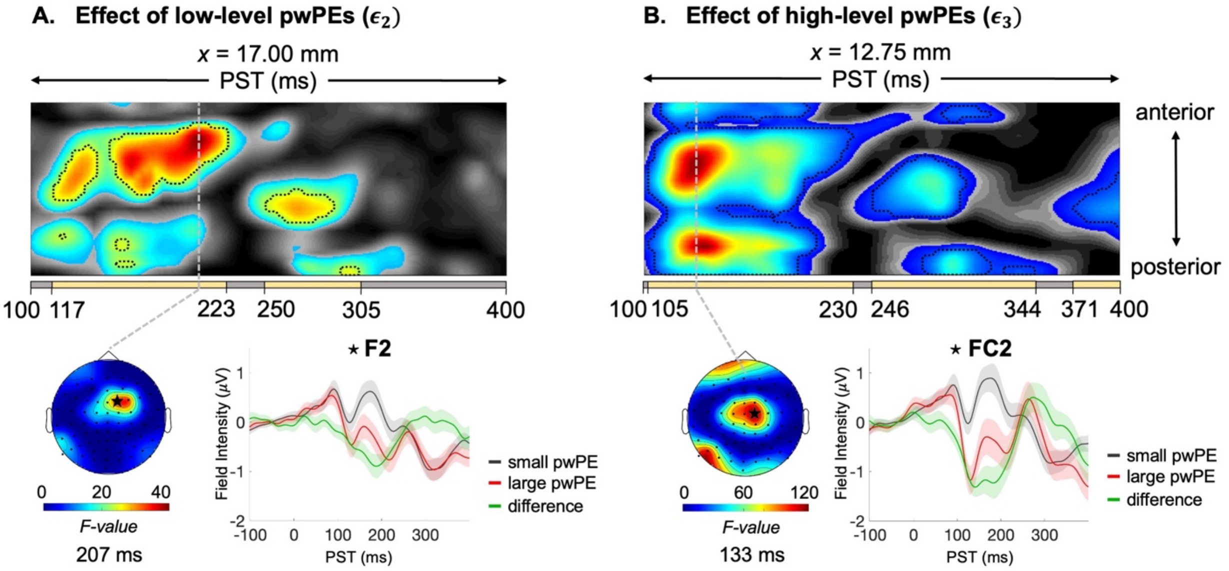

Figure S2: Precision-weighted prediction error (pwPE) expression. Maximum intensity projection *F*-map illustrating the effect of **(A)** low-level sensory pwPEs (ε_2) and **(B)** high-level volatility pwPEs (ε_3) on EEG amplitudes across anterior to posterior scalp locations (top). Significant peak-level effects ($p<0.05$, whole-volume FWE-corrected) are outlined by black contours, while the coloured area indicates *F*-values exceeding the cluster-defining threshold of $p<0.001$, uncorrected. The yellow bar at the bottom of the *F*-map indicates the time range of significant peak effects, from earliest to latest significant time points. On the left, the scalp map displays the peak effect of the given cluster using an *F*-map at the indicated PST, displayed on a 2D sensor layout. On the right, ERPs were averaged across the electrode at the peak of the significant clusters using the 10% largest and 10% smallest pwPE values. The selected electrode position indicated by a star on the scalp map. MMN: mismatch negativity; PST: peri-stimulus time; FWE: family wise error.

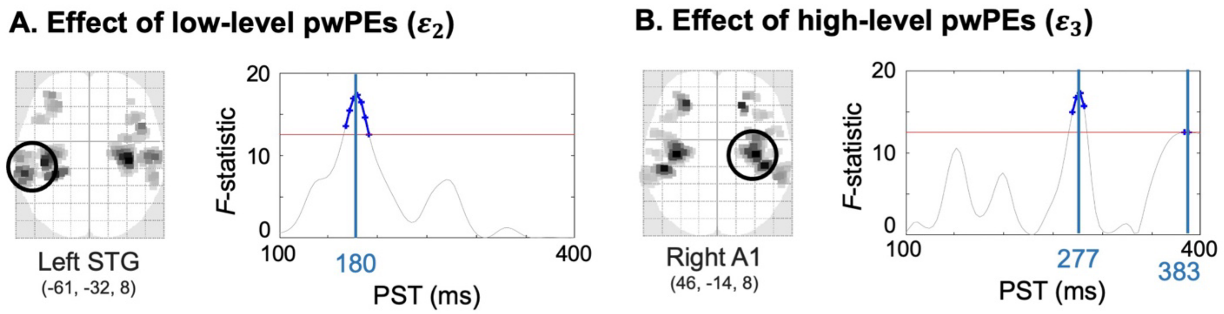

Figure S3: Cortical generators of precision-weighted prediction errors (pwPEs). **(A)** Source activation of low-level pwPEs, estimated from the grand-averaged difference waveform (10% highest - 10% lowest ε_2 trials) at the peak time point (180ms), is depicted using an SPM-glass brain (left). The neurological orientation was employed. Significant *F*-contrasts of the pwPE effect are presented for the left STG over peri-stimulus time (PST) (right). The significance threshold under peak-family wise error (FWE) correction is indicated by the red horizontal line, and all time points above this threshold are colored in blue. The peak time point is marked by the blue vertical line. **(B)** Source activation of high-level pwPEs in the right A1 peaked at 277ms and 383ms post-stimulus. pwPE: precision-weighted prediction error; PST: peri-stimulus time; GF: global function; A1: primary auditory cortex; STG: superior temporal gyrus.

Supplementary Tables

***Table S1**: Hierarchical Gaussian filter perceptual model parameters.*

Parameter	Prior	
	Mean	Variance
κ	log(1)	0
ω_2	-3	4
ω_3	2	4
$\mu_2^{k=0}$	0	0
$\sigma_2^{k=0}$	log(0.1)	0
$\mu_3^{k=0}$	1	0
$\sigma_3^{k=0}$	log(1)	0

***Table S2**: Hierarchical Gaussian filter ideal observer parameters.*

	Healthy Controls (n = 43)
ω_2	-1.91
ω_3	1.95

***Table S3:** Montreal Neurological Institute (MNI) coordinates of predefined sources.*

Source	Hemisphere	x	y	z
Primary auditory cortex	L	-42	-22	7
Primary auditory cortex	R	46	-14	8
Superior temporal gyrus	L	-61	-32	8
Superior temporal gyrus	R	59	-25	8
Inferior frontal gyrus	L	-46	-20	8
Inferior frontal gyrus	R	46	20	8

***Table S4**: Sensor-level summary statistics of significant clusters of activation for mismatch difference waveform.*

Mismatch response	Cluster size (voxels)	Cluster *p*-value (FWE-corrected)	Peak *p*-value (FWE-corrected)	F_{42} (peak value)	Peak location (mm, mm, ms)
stable MMN	3306	**<0.001**	**<0.001**	113.94	-17, 29, 141
	131	**0.001**	**<0.001**	46.82	-21, 72, 164
	134	**0.001**	**0.001**	39.28	0, -95, 309
	324	**<0.001**	**0.001**	38.36	17, -25, 281
	106	**0.001**	**0.002**	37.69	21, 72, 145
	146	**0.001**	**0.003**	35.28	-21, -73 133
	83	**0.003**	**0.005**	32.71	8, -89, 172
	27	**0.012**	**0.021**	27.37	42, -62, 137
volatile MMN	2605	**<0.001**	**<0.001**	88.00	0, 2, 266
	583	**<0.001**	**<0.001**	52.48	-51, -57, 277
	73	**0.003**	**0.007**	32.01	-38, -68, 148
	27	**0.011**	**0.011**	30.20	13, 72, 320
	21	**0.014**	**0.034**	25.70	17, 40, 145
	5	**0.031**	**0.037**	25.43	42, -3, 141
	4	**0.033**	**0.041**	25.05	38, 18, 141

Significant values (p<0.05) are shown in bold.

***Table S5**: Sensor-level summary statistics of significant clusters of activation for difference in stable and volatile mismatch difference waveform.*

Mismatch response	Cluster size (voxels)	Cluster *p*-value (FWE-corrected)	Peak *p*-value (FWE-corrected)	t_{42} (peak value)	Peak location (mm, mm, ms)
stable MMN > volatile MMN	551	**0.007**	**0.019**	5.08	42, -62, 145
stable MMN < volatile MMN	1649	**<0.001**	**0.002**	5.88	-30, -19, 152

Significant values (p<0.05) are shown in bold.

Table S6: *Sensor-level summary statistics for associations between global function measures and mismatch difference waveform.*

Mismatch parameter	Cluster size (voxels)	Cluster *p*-value (FWE-corrected)	Peak *p*-value (FWE-corrected)	t_{41} (peak value)	Peak location (mm, mm, ms)
**GF Role**					
**oddball MMN** pos. effect	350	**0.038**	**0.049**	4.65	-51, -30, 387
**stable MMN** pos. effect	297	**0.049**	0.113	4.33	-55, -25, 387
**GF Social**					
**oddball MMN** pos. effect	791	**0.003**	0.064	4.54	0, 13, 332
**volatile MMN** pos. effect	720	**0.003**	**0.036**	4.81	0, 24, 344

Significant values (p<0.05) are shown in bold.

Table S7: *Source-level summary statistics for mismatch difference waveform.*

Source	Cluster size (voxels)	Cluster *p*-value (FWE-corrected)	Peak *p*-value (FWE-corrected)	F_{42} (peak value)	Peak time (ms)
**Difference Waveform**					
Left A1	18	**<0.001**	**0.001**	21.27	246
Right A1	13	**<0.001**	**0.002**	17.89	281
**Difference Waveform: Volatile**					
Left A1	5	**0.007**	**0.007**	14.99	285

Significant values (p<0.05) are shown in bold.

Table S8: *Source-level summary statistics for associations between global function measures and mismatch difference waveform.*

Source	Cluster size (voxels)	Cluster *p*-value (FWE-corrected)	Peak *p*-value (FWE-corrected)	t_{41} (peak value)	Peak time (ms)
**GF Role**					

**stable MMN & left STG:** *neg. effect*	19	**<0.001**	**<0.001**	4.79	160

Significant values (p<0.05) are shown in bold.

Table S9: *Sensor-level summary statistics of significant clusters of activation for computational trajectories and mismatch difference waveform.*

Comput-ational parameter	Cluster size (voxels)	Cluster *p*-value (FWE-corrected)	Peak *p*-value (FWE-corrected)	F_{42} (peak value)	Peak location (mm, mm, ms)
ε_2	4675	**<0.001**	**0.001**	42.66	21, 40, 207
	1188	**<0.001**	**0.006**	32.11	4, -30, 266
	1147	**<0.001**	**0.029**	26.16	-60, -68, 156
	384	**0.019**	**0.030**	26.07	0, -95, 301
ε_3	8882	**<0.001**	**<0.001**	123.41	17, 13, 133
	3532	**<0.001**	**<0.001**	123.38	-51, -68, 137
	2048	**<0.001**	**<0.001**	57.78	0, -3, 281
	1149	**<0.001**	**<0.001**	55.14	0, -84, 293
	917	**0.001**	**<0.001**	53.30	-30, 67, 121
	495	**0.009**	**0.002**	37.66	60, -30, 398
	316	**0.025**	**0.012**	29.34	-47, -41, 398
	350	**0.022**	**0.036**	25.13	0, -3, 398
δ_1	10683	**<0.001**	**<0.001**	140.89	17, 18, 137
	4333	**<0.001**	**<0.001**	136.18	-42, -62, 141
	5594	**<0.001**	**<0.001**	83.12	4, -9, 277
	2497	**<0.001**	**<0.001**	76.20	0, -89, 297
	951	**0.001**	**<0.001**	60.13	-21, 72, 156
	481	**0.014**	**0.002**	34.94	-8, 8, 398
δ_2	10506	**<0.001**	**<0.001**	147.99	-17, 18, 141
	3952	**<0.001**	**<0.001**	131.39	-42, -73, 156
	5152	**<0.001**	**<0.001**	78.26	8, -19, 285

	2272	**<0.001**	**<0.001**	65.51	-21, -84, 285
	943	**0.001**	**<0.001**	56.60	-21, 72, 156
	558	**0.009**	**0.001**	38.20	-4, 8, 398
ψ_2	557	**0.005**	**0.015**	28.85	-17, -3, 117
	1304	**<0.001**	0.074	22.78	42, -9, 246
	257	**0.050**	0.110	21.27	42, 2, 188
	288	**0.038**	0.187	19.25	-60, -57, 219
ψ_3	2482	**<0.001**	**0.002**	36.33	13, 40, 207
	222	0.069	**0.011**	30.03	0, -95, 152
difference	4324	**<0.001**	**<0.001**	83.48	8, -3, 277
waveform	2239	**<0.001**	**<0.001**	79.35	-51, -57, 277
	5712	**<0.001**	**<0.001**	73.09	13, 29, 145
	1535	**<0.001**	**<0.001**	68.94	-34, -73, 141
	486	**0.011**	**<0.001**	44.68	4, 67, 320
	390	**0.021**	**0.001**	41.59	-30, 67, 168

Significant values (p<0.05) are shown in bold.

Table S10*: Source-level summary statistics of significant clusters of activation for computational parameters and mismatch difference waveform.*

Source	Cluster size (voxels)	Cluster *p*-value (FWE-corrected)	Peak *p*-value (FWE-corrected)	F_{42} (peak value)	Peak time (ms)
**low-level precision-weighted prediction error (ε_2)**					
Left A1	5	**0.006**	**0.012**	13.96	273
Left STG	5	**0.006**	**0.005**	16.49	180
**high-level precision-weighted prediction error (ε_3)**					
	6	**0.003**	**0.002**	18.99	273
Right A1	3	**0.013**	**0.013**	13.91	152
	9	**<0.001**	**0.016**	13.35	379
**low-level unweighted prediction error (δ_1)**					

Left STG	10	**<0.001**	**0.003**	17.36	152
**low-level unweighted prediction error ($\boldsymbol{\delta_2}$)**					
Left A1	8	**0.001**	**0.003**	18.32	172
Right A1	12	**<0.001**	**0.001**	22.41	398
	6	**0.003**	**0.007**	15.43	273
	2	**0.016**	**0.019**	12.65	188
Left STG	14	**<0.001**	**0.001**	21.23	180
**high-level precision ratio ($\boldsymbol{\psi_3}$)**					
Left A1	1	**0.022**	**0.022**	12.75	266

Significant values (p<0.05) are shown in bold.

Table S11: *Source-level summary statistics for associations between global function measures and computational trajectories.*

Source	Cluster size (voxels)	Cluster p-value (FWE-corrected)	Peak p-value (FWE-corrected)	t_{41} (peak value)	Peak time (ms)
**GF Role**					
**$\boldsymbol{\delta_1}$ & right STG:** *neg. effect*	12	**<0.001**	**<0.001**	5.20	156
**$\boldsymbol{\delta_2}$ & right STG:** *neg. effect*	9	**0.001**	**0.001**	4.35	156

Significant values (p<0.05) are shown in bold.

Table S12: *Source-level summary statistics for associations between covariates and both computational trajectories and oddball responses.*

Source	Cluster size (voxels)	Cluster p-value (FWE-corrected)	Peak p-value (FWE-corrected)	F_{41} (peak value)	Peak time (ms)
**Age**					
oddball volatile & right A1:	4	**0.010**	**0.002**	18.71	160
	12			17.82	320
	2	**<0.001**	**0.003**	13.77	199

		**0.014**	**0.011**		
oddball volatile & left STG:	6	**0.007**	**0.001**	20.02	320
oddball volatile & right STG:	10	**0.003**	**0.003**	16.56	320
ψ_2 & left IFG:	2	**0.018**	**0.005**	15.49	102
ψ_3 & left IFG:	1	**0.021**	**0.018**	13.10	297
**Working Memory**					
Oddball & right STG	3	**0.012**	**0.012**	13.23	312

Significant values (p<0.05) are shown in bold.

Supplementary References

1. Adams, R. A., Pinotsis, D., Tsirlis, K., Unruh, L., Mahajan, A., Horas, A. M., Convertino, L., Summerfelt, A., Sampath, H., Du, X. M., Kochunov, P., Ji, J. L., Repovs, G., Murray, J. D., Friston, K. J., Hong, L. E., & Anticevic, A. (2022). Computational Modeling of Electroencephalography and Functional Magnetic Resonance Imaging Paradigms Indicates a Consistent Loss of Pyramidal Cell Synaptic Gain in Schizophrenia. *Biological Psychiatry*, 91(2), 202–215. https://doi.org/10.1016/j.biopsych.2021.07.024
2. Charlton, C. E., Lepock, J. R., Hauke, D. J., Mizrahi, R., Kiang, M., & Diaconescu, A. O. (2022). Atypical prediction error learning is associated with prodromal symptoms in individuals at clinical high risk for psychosis. *Schizophrenia, 8*(1), 105. https://doi.org/10.1038/s41537-022-00302-3
3. Garrido, M. I., Friston, K. J., Kiebel, S. J., Stephan, K. E., Baldeweg, T., & Kilner, J. M. (2008). The functional anatomy of the MMN: a DCM study of the roving paradigm. *NeuroImage, 42*(2), 936–944. https://doi.org/10.1016/j.neuroimage.2008.05.018
4. Garrido, M. I., Kilner, J. M., Kiebel, S. J., & Friston, K. J. (2009). Dynamic causal modeling of the response to frequency deviants. *Journal of Neurophysiology, 101*(5), 2620–2631. https://doi.org/10.1152/jn.90291.2008
5. Jardri, R., & Denève, S. (2013). Circular inferences in schizophrenia. *Brain, 136*(Pt 11), 3227–3241. https://doi.org/10.1093/brain/awt257
6. Kass, R. E., & Raftery, A. E. (1995). Bayes factors. *Journal of the American Statistical Association, 90*(430), 773–795. https://doi.org/10.1080/01621459.1995.10476572

7. Nolte, G., & Hämäläinen, M. S. (2001). Partial signal space projection for artefact removal in MEG measurements: a theoretical analysis. *Physics in Medicine and Biology, 46*(11), 2873–2887. https://doi.org/10.1088/0031-9155/46/11/308
8. Phillips, H. N., Blenkmann, A., Hughes, L. E., Bekinschtein, T. A., & Rowe, J. B. (2015). Hierarchical Organization of Frontotemporal Networks for the Prediction of Stimuli across Multiple Dimensions. *The Journal of Neuroscience, 35*(25), 9255–9264. https://doi.org/10.1523/JNEUROSCI.5095-14.2015
9. Stephan, K. E., Penny, W. D., Daunizeau, J., Moran, R. J., & Friston, K. J. (2009). Bayesian model selection for group studies. *NeuroImage, 46*(4), 1004–1017. https://doi.org/10.1016/j.neuroimage.2009.03.025
10. Weber, L. A., Diaconescu, A. O., Mathys, C., Schmidt, A., Kometer, M., Vollenweider, F., & Stephan, K. E. (2020). Ketamine Affects Prediction Errors about Statistical Regularities: A Computational Single-Trial Analysis of the Mismatch Negativity. *The Journal of Neuroscience, 40*(29), 5658–5668. https://doi.org/10.1523/JNEUROSCI.3069-19.2020
11. Wechsler, D. (1981). *Wechsler adult intelligence scale-revised* (*WAIS-R*). Psychological Corporation, San Antonio.
